# Glaucoma in a Patient with Nanophthalmos

**Published:** 2011-07

**Authors:** Kouros Nouri-Mahdavi, Naveed Nilforushan, Mohammad-Reza Razeghinejad, Heydar Amini, Shamira A Perera

**Affiliations:** Assistant Professor, Jules Stein Eye Institute, UCLA, Los Angeles, California, USA; Assistant Professor, Eye Research Center, Tehran University of Medical Sciences, Tehran, Iran; Associate Professor, Shiraz University of Medical Sciences, Shiraz, Iran; Professor, Tehran University of Medical Sciences, Tehran, Iran; Consultant, Singapore National Eye Center, Singapore

## CASE PRESENTATION

The patient presented herein is a 34-year-old woman with nanophthalmos and secondary glaucoma. Her mental ability is mildly impaired. She has no history of systemic disorders and is not receiving any systemic medications except acetazolamide 250 mg every 6 hours. She has history of bilateral peripheral laser iridectomies, phacoemulsification with implantation of a posterior chamber intraocular lens (IOL) in the right eye, followed by an anterior chamber Artisan lens, 5 years before presentation. The power of the IOLs was not available. Glaucoma had been diagnosed in both eyes since 2 years before.

**Figure 1:**
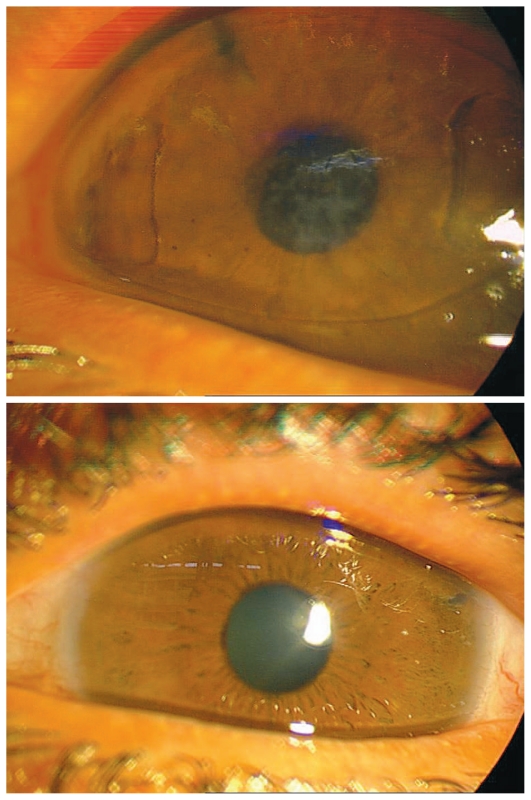
Slit lamp photographs of the right (top) and left eyes (bottom) at presentation.

Initial examination upon presentation was as follows: best corrected visual acuity was 1/10 in the right eye with +12, and 6/10 in the left eye with +12−1.5×150°. Slit lamp examination of the right eye revealed mild conjunctival injection, mild corneal haziness and stromal edema, and a deep anterior chamber with a centered Artisan lens in touch with the corneal endothelium peripherally. A peripheral iridectomy was present and some posterior synechiae were noted between the pupillary margin and the posterior chamber IOL. Moderate posterior capsule opacity was also evident. In the left eye the cornea was clear, the anterior chamber was shallow and quiet, there was a peripheral iridectomy and the crystalline lens was clear (Fig. 1). On gonioscopy, the right eye had 360 degrees of peripheral anterior synechiae (PAS), and the left eye had more than 300 degrees of PAS. Intraocular pressure (IOP) was 36 and 30 mmHg in the right and left eyes respectively with Xalatan 0.005% (latanoprost) daily, Cosopt (dorzolamide 2%/timolol 0.5%) twice a day, brimonidine 0.02% three times a day in both eyes, and acetazolamide 250 mg every 6 hours. Central corneal thickness (CCT) was 610 and 585 microns in the right and left eyes respectively. Fundus examination revealed a vertical cup/disc ratio of 0.5 bilaterally, in small crowded discs (vertical disc diameters, 1.2 mm) which were moderately pale. The fundus examination was otherwise unremarkable.

Perimetry was attempted a couple of times but was totally unreliable with a clover leaf pattern in both eyes. Ocular biometric, ultrasonic biomicroscopic (UBM), and Pentacam measurements were obtained and are summarized in table 1. We were unable to capture high quality fundus or optical coherence tomography (OCT) images. Anterior visual pathway MRI was performed which was normal.

What would you recommend regarding the management of this patient?

### Kouros Nouri-Mahdavi, MD

The patient is a 34-year old female with nanophthalmos and mild mental impairment. She has no known systemic disorders and past ocular history is positive for presumably uncomplicated phacoemulsification with placement of a posterior chamber IOL in the right eye and seemingly secondary implantation of an Artisan iris-claw lens and bilateral peripheral laser iridectomies. Glaucoma developed subsequently with peripheral lens-cornea touch and posterior synechiae in the right eye. The angle is closed for the most part in both eyes. IOP is running in the 30s in both eyes on maximal treatment, with a fairly thick CCT. The patient has small discs with significant disc damage and mild pallor in both eyes, and a reliable visual field cannot be obtained. Based on the biometry, the eyes are very small. I assume that the reported anterior chamber depth (ACD) represents actual ACD and does not include corneal thickness.

This is indeed a very challenging case. The right eye needs to be addressed first because of higher IOP and evolving corneal edema. In addition to glaucoma management, refractive correction is an issue that requires a thorough discussion with the patient preoperatively. I assume the patient was extremely hyperopic after the phacoemulsification and hence, an Artisan lens was secondarily placed. At this point, the Artisan lens needs to be removed before the situation gets more complicated. Since the patient is bound to have corneal surgery at some point, a glaucoma drainage device is a potentially good choice. The eye is, however, very small and only a small or pediatric size implants (such as a single plate Molteno or pediatric Ahmed glaucoma valve) would be appropriate, with possibly less efficacy than desired. If the superior conjunctiva is untouched, my first choice for glaucoma surgery would be a trabeculectomy with mitomycin-C (MMC). I would remove the Artisan lens and create inferior scleral windows on the first surgical session before proceeding with trabeculectomy in the right eye. Nanophthalmic eyes can get into trouble after any type of drainage surgery because of poor drainage of the vortex veins and very thick sclera. Obviously, the patient would end up extremely hyperopic again. Unfortunately, there are no good options available for her at this point. A piggyback lens would be asking for more trouble since the anterior chamber cannot accommodate any further volume. I would also be reluctant to consider IOL exchange. We may have to settle for contact lenses postoperatively although with her steep corneas this may also be problematic. I would obtain a refractive surgery consultation before any intervention to see if any rarely used corneal surgical option such as epikeratophakia would be appropriate in this particular case. If the trabeculectomy fails in the right eye, one could always resort to a tube. For the left eye, given the history of uncomplicated phacoemulsification in the right eye, I would suggest removal of the crystalline lens with inferior scleral windows on the first surgical session with subsequent trabeculectomy after 2 to 3 months. It is now possible to find IOLs in very high powers (> 40 D), which may be appropriate for the left eye. Alternatively, a customized IOL with appropriate power could be ordered while she is having surgery in her right eye. Obviously, IOL power would depend on availability and final correction in the right eye. One possibility would be to relinquish improving refractive correction in the right eye and concentrate on getting the best possible correction on the left.

One final note is that given the high risk of complications following any type of intraocular surgery, all the risks and benefits of intervention should be discussed with the patient before proceeding.

### Naveed Nilforushan, MD

Management of eyes with nanophthalmos is always a dilemma for the ophthalmologist, especially if the condition is associated with glaucoma. The main concern with any type of penetrating surgery in these patients is the high risk of sight-threatening intra- and postoperative complications. These include but are not limited to malignant glaucoma, choroidal and retinal detachment, suprachoroidal hemorrhage, flat anterior chamber and corneal decompensation. A detailed history of previous surgery on the fellow eye and its complications could help make a better decision for the other eye. In nanophthalmic eyes, a diagnosis of glaucoma and its progression is often complicated. The disc is small, so even slight cupping could be a sign of glaucoma. Visual field testing in patients with high plus lenses and low vision (due to amblyopia or posterior pole folds from uveal effusion) can be misleading and inaccurate. In this young patient with uncontrolled angle closure glaucoma on maximum medical therapy, surgery would be the only choice. In general, before any intraocular procedure in nanophthalmic eyes, UBM and posterior segment B scan should be performed. These tests can provide valuable information regarding the presence of supraciliary and choroidal effusion, and choroidal and scleral thickness. In this particular case and for the right eye with corneal edema, an Artisan IOL and high IOP my suggestions are:

1. Surgery under general anesthesia with 20% mannitol serum injection before starting the procedure.
2. If there is choroidal thickening or any suspicion of uveal effusion on B-scan or fundus examination, I would first perform an anterior sclerotomy in both inferior quadrants.
3. The next step would be anterior chamber IOL removal via a corneolimbal incision. Injection of cohesive viscoelastics, such as Healon GV and Healon 5, before IOL removal maintains ACD and reduces the risk of endothelial damage, uveal effusion and aqueous misdirection.
4. If everything goes well and the conjunctiva is sufficient with only slight scarring from previous surgery, MMC augmented trabeculectomy with tight scleral flap sutures can be performed. Otherwise, a small size Ahmed valve (model FP8 or S3) can be implanted. If both of the above-mentioned procedures are not feasible, limited endoscopic cyclophotocoagulation (not more than 180°), using low laser power and long duration would be my next option.
5. Atropinizing the eye and subconjunctival injection of corticosteroids should be performed at the end of surgery.
6. Systemic corticosteroids should be prescribed a few days before and after surgery. This may decrease the risk of uveal effusion or help resolve subtle undetectable existing effusion.

In the left eye, for better control of IOP and prevention of progressive angle closure, lens aspiration and implantation of a foldable posterior chamber IOL together with viscogoniosynechialysis is recommended. Based on preoperative refraction and IOL calculation, it seems that the lens is more or less spherical. Thus, removing the lens will probably deepen the anterior chamber angle significantly. I prefer to implant a single lens in the bag (with highest available power, +40) and have some residual refractive error rather than use piggyback IOLs with possible complications of interlenticular opacity, decentration, pigment dispersion and hyperopic shift. For the left eye, the same considerations mentioned for the right eye should be taken into account. In addition, we have to keep in mind that any manipulation may be associated with disastrous intra- and postoperative complications.

### Mohammad-Reza Razeghinejad, MD

The presented patient is a case of nanophthalmos with the following issues:

1. Uncontrolled IOP.
2. Progressive corneal endothelial damage in the right eye due to contact with the IOL.
3. Refractive error.
4. Progressive PAS formation in the left eye.

These are discussed individually below:

#### 1. Uncontrolled IOP

The patient is receiving maximum medical therapy and still has uncontrolled IOP. The Goldmann tonometer is assumed to measure IOP accurately when CCT is 520 microns and many correction formulas have been introduced for thicker and thinner corneas. Although almost all reports agree that CCT affects IOP measurement, there is no consensus regarding a specific formula for IOP correction in routine clinical practice. In addition to CCT, corneal biomechanics also affect measured IOP.

The level of IOP in this particular patient is high and surgical intervention is necessary, despite the fact that surgery in nanophthalmic eyes has an extremely high complication rate with potentially disastrous results. Singh et al reported that 60% of 15 nanophthalmic patients undergoing filtration surgery for glaucoma failed to achieve control and 86.6% sustained visual loss.

Regarding the high rate of choroidal effusion or hemorrhage and exudative retinal detachment in nanophthalmic eyes following any kind of surgery including laser iridotomy, it is necessary to make prophylactic sclerostomies in both inferior quadrants before creating any opening into the eye. Trabeculectomy with antifibrotics and tight releasable sutures can be employed, but this may still be associated with early postoperative hypotony resulting in choroidal effusion or hemorrhage. I would prefer a Molteno tube implantation; tube ligature with Vicryl sutures will prevent early postoperative hypotony. After 4 to 6 weeks, when a fibrous capsule has formed around the shunt plate, the Vicryl suture dissolves and IOP reduction occurs gradually. Considering the short axial length, if the available shunt is Ahmed, I would rather trim the posterior third of the plate (FP7) to prevent possible optic nerve compression. Based on the “Trabeculectomy versus Tube” trial, IOPs in the low teens are achievable with shunts, as attained in 62% of patients in the tube group at 3 years.

#### 2. Progressive corneal endothelial damage

There is no doubt that the Artisan lens should be explanted because of contact with the cornea and progressive endothelial damage. Before performing any type of intraocular surgery I would prefer to obtain a specular microscopy and discuss the possibility of corneal decompensation with the patient if the cell count is low.

#### 3. Refractive error

To correct the refractive error in the right eye, a three-piece IOL can be implanted in the sulcus after removing the Artisan lens. Power calculation for piggyback IOLs is independent of axial length but relies on careful manifest refraction. For eyes with hyperopic errors up to 7 D, the spherical equivalent (SE) can be multiplied by 1.5. For myopic errors up to 7 D, SE can be adjusted by a factor of 1.3. In eyes with a power surprise outside the ±7 D range, or if increased accuracy is sought, the Holladay R formula (refractive vergence formula) can be used which is available at: http://doctor-hill.com/physicians/download.htm#four.

Although there is a space between the two implanted lenses (Artisan and posterior chamber IOL) which may affect the refractive error and consequently, measured piggyback IOL power, the two mentioned formulas seem to be the only available methods for determining piggyback IOL power in this patient. In order to calculate piggyback IOL power in the right eye using the refractive vergence formula, we need to know K1, K2, effective lens position (calculated by subtracting 0.65 mm from the IOL manufacturer’s bag ACD), and vertex distance. With the available data, I cannot calculate the power of the piggyback IOL. The left eye can receive in the bag and piggyback IOLs after lens extraction.

#### 4. Progressive PAS formation in the left eye

Patients with nanophthalmic eyes have normal or thick lens and high lens-to-eye volume ratio. This ratio, which is about 4% in normal eyes, is increased up to 10 to 30% in nanophthalmos. Aging is associated with an increase in lens thickness which makes the iridocorneal angle narrower and PAS more extensive. To prevent PAS formation at the open portion of the angle and release existing PAS, phacoemulsification and IOL implantation combined with viscogoniosynechialysis seems to be an effective procedure. If this procedure cannot control the IOP, filtering surgery is necessary but considering probable complications, I would prefer a shunt procedure.

All in all, my surgical plan for this patient would include Artisan lens explantation, piggyback IOL implantation, shunt surgery, and prophylactic sclerostomies for the right eye; and phacoemulsification, IOL implantation, viscogoniosynechialysis, and prophylactic sclerostomies in the left eye. If IOP is still not controlled in the left eye, my next recommendation would be shunt surgery.

Suggested Readings1BashfordKPShafranovGTauberSShieldsMBConsiderations of glaucoma in patients undergoing corneal refractive surgerySurv Ophthalmol2005502452511585081310.1016/j.survophthal.2005.02.0062SinghOSSimmonsRJBrockhurstRJTrempeCLNanophthalmos: a perspective on identification and therapyOphthalmology1982891006101271775653GeddeSJSchiffmanJCFeuerWJHerndonLWBrandtJDBudenzDLTube Versus Trabeculectomy Study GroupThree-year follow-up of the tube versus trabeculectomy studyAm J Ophthalmol20091486706841967472910.1016/j.ajo.2009.06.0184HillWEByrneSFComplex axial length measurements and unusual IOL power calculationsFocal Points: Clinical Modules for Ophthalmologists, Module 22San FranciscoThe American Academy of Ophthalmology20045RazeghinejadMRRahatFCombined phacoemulsification and viscogoniosynechialysis in the management of patients with chronic angle closure glaucomaInt Ophthalmol2010303533592017795510.1007/s10792-010-9353-4

## Heydar Amini, MD

This is a case of nanophthalmos in a young woman complicated by secondary glaucoma; however, other diagnoses such as simple and posterior microphthalmos may be considered. Nanophthalmic patients have small corneal diameter, while those with posterior microphthalmos have normal-sized corneas. In addition, other ocular anomalies, especially in the posterior segment are typical of complex microphthalmos. The patient has slight mental disability, which is the case in one-fifth of nanophthalmic patients.

The Artisan IOL is in touch with the peripheral corneal endothelium in the right eye and this could result in progressive corneal decompensation and synechiae formation. The patient has corneal edema which denotes compromised endothelial function. Knowledge of endothelial cell counts would aid in decision making. I am dubious about the initial decision for implanting an Artisan IOL, since this procedure is contraindicated in eyes with shallow anterior chamber (ACD less than 2.7 mm, measured from the endothelium). ACD in the right eye was reported to be 2.57 mm. However, if the measurement is performed by ultrasonic methods, it is representative of ACD measured from the epithelium. Considering a CCT of 610 microns, the endothelial ACD should be about 1.96 mm, which is far less than the recommended safe limit. With these considerations, I believe that removal of the Artisan IOL is necessary. I need an endothelial cell count to decide on the next surgical step. There are two major clinical scenarios in this situation. If there is sufficient endothelial reserve and the corneal edema is due to a “stressed” endothelium, I would proceed with implantation of a piggyback IOL. The posterior synechiae should be released during this procedure. In the more probable clinical situation of decreased endothelial reserve, I would remove the Artisan IOL and postpone additional refractive correction. There is evidence that in this situation, endothelial cells may repopulate or at least undergo redistribution after removal of the offending IOL.

The eye is not quiet and there is a high rate of failure in such cases. Before surgery, I would prescribe a short course of topical steroids and tear substitutes to decrease conjunctival hyperemia. I would perform goniosynechialysis during Artisan removal; this can decrease intraocular pressure in some patients. If IOP does not fall to the desired level, my first choice for glaucoma management in the right eye would be an aqueous drainage device (ADD) and I would choose a small Ahmed glaucoma valve (FP8 Ahmed glaucoma valve; New World Medical Inc., Rancho Cucamonga, CA, USA). To further decrease the risk of sudden ocular decompression I would fill the anterior chamber with viscoelastic agents and ligate the tube. If the patient has sufficient endothelial count, I would place the tube into the anterior chamber and reserve the ciliary sulcus for piggyback IOL. On the other hand, if endothelial count is critically low, I would insert the tube into the ciliary sulcus to prevent further endothelial damage. By this means, prophylactic sclerotomies are not obligatory; however, in high-risk patients it would be preferable to have anterior sclerotomies in the inferior quadrants.

Other modalities such as endoscopic cyclophotocoagulation (ECP) could also be used; however, the surgeon should be cautious about possible complications. In the past decade, I have attempted trans-scleral cyclophotocoagulation in a number of nanophthalmic patients with advanced glaucoma. Unfortunately, there was an unacceptably high rate of complications in these patients, resulting in phthisis bulbi.

In the left eye, after ruling out ciliary effusion and the possibility of malignant glaucoma following laser iridotomy, I would proceed to argon laser peripheral iridoplasty (ALPI). This could alleviate some of the appositional angle closure and decrease IOP to a safe level. If IOP remains uncontrollable, my next step would be phacoemulsification and viscogoniosynechialysis. One could create prophylactic sclerotomies to avoid intraoperative complications. In refractory cases, one could proceed to trabeculectomy with MMC and inferior sclerotomies, or alternatively, ADD, as described for the right eye.

Suggested Readings1CaoKYSitMBraga-MeleRPrimary piggyback implantation of 3 intraocular lenses in nanophthalmosJ Cataract Refract Surg2007337277301739775010.1016/j.jcrs.2006.11.0282HanLCairnsJDNanophthalmos with longstanding choroidal effusion and serous retinal detachmentAust N Z J Ophthalmol199725181183926761010.1111/j.1442-9071.1997.tb01305.x3HuangSYuMQiuCYeTThe management of secondary glaucoma in nanophthalmic patientsYan Ke Xue Bao200218156159155107454KhanAOPosterior microphthalmos versus nanophthalmosOphthalmic Genet2008291891900599310.1080/138168108022588625NunesCMMcNeillMPTongMGKuntz NavarroJBSpaethGLDorzolamide as an Effective Topical Therapy for Glaucoma in a Case of NanophthalmosOphthalmic Surg Lasers Imaging2010[Epub ahead of print]10.3928/15428877-20100210-16203373696SchmollCDevlinHFosterPUveal Effusion Syndrome as a complication of cyclodiode therapy in nanophthalmos glaucomaEye (Lond)2011259639642145524010.1038/eye.2011.67PMC31781607SrinivasanSBatterburyMMarshIBFisherACWilloughbyCKayeSBCorneal topographic features in a family with nanophthalmosCornea2006257507561707767510.1097/01.ico.0000220770.19402.508ThapaSSPaudyalGChoroidal effusion following laser peripheral iridotomy for the treatment of angle closure glaucoma in a patient with nanophthalmosNepal Med Coll J200578182162957329VermaASFitzpatrickDRAnophthalmia and microphthalmiaOrphanet J Rare Dis20072471803939010.1186/1750-1172-2-47PMC224609810WalshMKGoldbergMFAbnormal foveal avascular zone in nanophthalmosAm J Ophthalmol2007143106710681752478610.1016/j.ajo.2007.01.05111WladisEJGewirtzMBGuoSCataract surgery in the small adult eyeSurv Ophthalmol2006511531611650021510.1016/j.survophthal.2005.12.00512YalvacISSatanaBOzkanGEksiogluUDumanSManagement of glaucoma in patients with nanophthalmosEye (Lond)2008228388431729378410.1038/sj.eye.670274213YuzbasiogluEArtunayOAgachanABilenHPhacoemulsification in patients with nanophthalmosCan J Ophthalmol2009445345391978958810.3129/i09-142

## Shamira A Perera, MBBS, BSc, FRCOphth

In summary, this is a 34-year-old nanophthalmic woman with extensive, bilateral synechial angle closure. She has uncontrolled IOP in both eyes, despite maximal medical therapy. Additionally, she has early corneal decompensation in her right eye secondary to an iris clip lens.

The diagnosis and management of glaucoma in this case is challenging. The thick CCT overestimates Goldmann IOP, the small diameter disc may belie marked retinal nerve fiber layer (RNFL) loss, and the inability to perform visual field testing robs the clinician of a functional test for monitoring progression. In such cases a spectral domain OCT may detect subtle wedge-shaped RNFL defects, Heidelberg retinal tomography may monitor deterioration of disc parameters, and stereophotographs could offer reliable, contemporaneous documentation of disc morphology.

Nanophthalmic eyes have significant choroidal congestion secondary to impaired vortex venous drainage through the characteristically thickened sclera. They also have a high lens/eye volume ratio. Because of potentially blinding complications such as uveal effusion and serous retinal detachment associated with surgical intervention in these eyes, it is prudent to counsel the patient thoroughly.

In preparation, a preoperative UBM can seek the presence of subclinical choroidal effusion, primarily to predict the risk of effusion-related complications, but also for any superimposed lens subluxation.

To control IOP in the left eye, my approach would be a phacoemulsification with IOL implantation and trabeculectomy with MMC (0.2 mg/ml for 2 minutes) under peribulbar anesthesia. Preoperative intravenous mannitol infusion and prophylactic partial thickness sclerectomies and sclerostomies placed inferiorly would play an important role in ensuring safe surgery and a desirable outcome. Having said this, previous cataract surgery in the right eye had been uneventful and there was no note of uveal effusion in the history. Preplaced sutures, utilizing an anterior chamber maintainer and use of viscoelastics in the eye at the end of surgery could be an alternative approach to sclerotomy/sclerectomy. Tight flap sutures would ensure slow titration of IOP to an adequate level in the postoperative phase.

Some may argue that lens extraction is unnecessary considering the satisfactory visual acuity of 6/10. However, nanophthalmic eyes are predisposed to potential complications such as malignant glaucoma and suprachoroidal hemorrhage. These complications are more amenable to procedures such as hyaloidotomy if the eye is pseudophakic. Furthermore, this offers a refractive solution to the +12 hyperopia. Finally, cyclophotocoagulation is another option if the patient considers it too risky to perform surgery on their only eye.

My preferred management for the right eye would be to explant the iris clip lens very soon. This would be combined with a trabeculectomy augmented with MMC, utilizing the same precautions discussed earlier. An endothelial cell count would be useful in planning any further visual rehabilitation, either by a piggyback lens in the sulcus or a subsequent corneal graft if there is significant corneal decompensation.

Suggested Readings1Duke-ElderSAnomalies in the size of the eyeDuke-ElderSSystem of Ophthalmology3St. LouisCV Mosby19644882KimbroughRLTrempeCSBrockhurstRJSimmonsRJAngle-closure glaucoma in nanophthalmosAm J Ophthalmol19798857257911405710.1016/0002-9394(79)90517-83JohnsonMWGassJDSurgical management of the idiopathic uveal effusion syndromeOphthalmology199097778785237468210.1016/s0161-6420(90)32511-34YalvacISSatanaBOzkanGEksiogluUDumanSManagement of glaucoma in patients with nanophthalmosEye (Lond)2008228388431729378410.1038/sj.eye.6702742

## Figures and Tables

**Table 1:** Major ocular biometric, ultrasonic biomicroscopic and Pentacam data

	Right eye	Left eye
Axial length	15.3 mm	15.7 mm
Anterior chamber depth	2.57 mm	1.57 mm
IOL power calculation by Hoffer Q Formula	57 diopters	55 diopters
Sulcus to sulcus diameter by UBM	9 mm	9 mm
Mean keratometry	50.9 diopters	50.2 diopters
Anterior chamber volume	87 mm^3^	62 mm^3^
Anterior chamber angle	45°	19.2°

IOL, intraocular lens; UBM, ultrasonic biomicroscopy
